# Methodological considerations in personalized methods for PEEP optimization with electrical impedance tomography

**DOI:** 10.1186/s13613-024-01288-0

**Published:** 2024-04-20

**Authors:** Inéz Frerichs, Tobias Becher, Zhanqi Zhao

**Affiliations:** 1 Department of Anesthesiology and Intensive Care Medicine, University Medical Center, Schleswig-Holstein, Campus Kiel, Kiel, Germany; 2 School of Biomedical Engineering, Guangzhou Medical University, Guangzhou, China; 3 Department of Critical Care Medicine, Peking Union Medical College Hospital, Beijing, China; 4 Institute of Technical Medicine, Furtwangen University, Villingen-Schwenningen, Germany

## Dear editor

We have read with interest the recently published article by Pavlovsky and colleagues [1]. The authors examined patients with ARDS during a decremental PEEP trial performed with steps of 3 cmH_2_O from 20 to 5 cmH_2_O. Airway and esophageal pressures as well as electrical impedance tomography (EIT) data were recorded simultaneously. Four different approaches were then applied to identify the ’optimum PEEP’ value from the recordings of each patient by determining those PEEP levels of the PEEP trial at which (1) the plateau pressure was 28–30 cmH_2_O, (2) the positive expiratory transpulmonary pressure reached its minimum (’positive P_L_E’), (3) the center of ventilation (CoV) was closest to 0.5 and (4) the relative lung overdistension and collapse curves crossed (’crossing point’). The latter two values were derived from the EIT data. With the exception of the ’crossing point’ and ’positive P_L_E’, the calculated ’optimum PEEP’ values varied significantly from each other, and none of the values was correlated to the recruitment-to-inflation ratio (R/I_est_), as calculated from the change in end-expiratory lung impedance between PEEP of 5 and 15 cmH_2_O.

In physiological studies investigating different parameters for individualization of PEEP settings, correct understanding and interpretation of the examined parameters is of utmost importance. Regarding the R/I_est_ parameter, a previous study suggested that its absolute value does not correlate with actual lung recruitment because this parameter cannot distinguish atelectasis and overdistension [2]. In the current study by Pavlovsky and colleagues [1], the ’optimum PEEP’ levels determined with the four abovementioned methods were not associated with R/I_est_, leading the authors to the conclusion that ”optimal PEEP levels proposed by these four methods were not associated with recruitability”. This conclusion may be inaccurate because R/I_est_ ignores overdistension and can be misleading. Since R/I_est_ is certainly not the gold standard for PEEP setting, as implicitly presented by the authors, it would be interesting to see if any of the other parameters were correlated with respect to ’optimum PEEP’ (e.g., was there a positive correlation between the ’positive P_L_E’ and ’crossing point’ PEEP values? ).

Besides these general methodological considerations, we would like to point out some important inaccuracies in the assessment of the EIT-derived CoV parameter. The top three panels of Fig. 1 show the individual patient values of CoV, relative overdistension and collapse. Surprisingly, the highest CoV values were identified at the lowest PEEP value of 5 cmH_2_O. The authors wrote in the Methods that CoV was ”the percentage of ventilation reaching the dorsal half of the lung”. The dorsal fraction of ventilation should not be the highest at the lowest PEEP level when derecruitment is most likely to have occurred in the dependent lung regions. The presented CoV values also contradict the findings presented in the top right panel showing the highest percentage of lung collapse at the lowest PEEP. The presented results would only make sense if the patients were examined in the prone position, however, the authors wrote that the patients were studied in the semi-recumbent position.

In addition to these physiologically implausible results, the authors also disregarded that CoV and the dorsal fraction of ventilation are two separate, not mutually interchangeable EIT parameters [3]. They are calculated differently and they provide dissimilar information on ventilation distribution. CoV is determined as the weighted geometrical center of ventilation distribution and its location is projected on the ventrodorsal axis of the chest and given in percent of chest diameter. Values lower than 50% imply more ventral and values higher than 50% more dorsal location. The dorsal fraction of ventilation, however, gives just the proportion of ventilation identified in the dorsal half of the analyzed functional EIT image. The Consensus Statement on Chest EIT [4] (which the authors cite in their article´s reference list) provides a clear description of the EIT measures and recommendations for unified use and reporting. If such recommendations are not adopted, the readers may become confused and the users of EIT technology could even draw wrong conclusions on the performance of EIT parameters.

Previous studies indicated that the dorsal fraction of ventilation showed large variation in lung-healthy patients and CoV was mostly located in the ventral part of the chest [5]. Based on these findings, the authors’ decision to define the ’optimum PEEP’ as the value when CoV location was at exactly 50% of the ventrodorsal chest diameter may not be a physiologically sound approach. Indeed, the authors found that CoV reached the value close to 50% of the chest diameter only at a PEEP value of 20 cmH_2_O. Since the overdistension most probably occurred mainly in the non-dependent ventral lung regions, this effect ’pushed’ the CoV towards the center of the chest. We therefore hypothesize that lower (i.e., more ventrally located) CoV values might be more appropriate for setting PEEP in future studies or clinical use.

## Data Availability

Not applicable.

## Abbreviations

ARDS: Acute respiratory distress syndrome
CoV: Center of ventilation
EIT: Electrical impedance tomography
P_L_E: Expiratory transpulmonary pressure
PEEP: Positive end-expiratory pressure
R/I_est_: Recruitment-to-inflation ratio
